# Research into Kinect/Inertial Measurement Units Based on Indoor Robots

**DOI:** 10.3390/s18030839

**Published:** 2018-03-12

**Authors:** Huixia Li, Xi Wen, Hang Guo, Min Yu

**Affiliations:** 1 Institute of Space Science and Technology, Nanchang University, Nanchang 330031, China; hxli0601@gmail.com (H.L.); wuhuawujiu@163.com (X.W.); 2 School of Resources Environment & Chemical Engineering, Ministry of Education Key Laboratory of Poyang Lake Environment and Resource Utilization, Nanchang University, Nanchang 330031, China; 3 College of Computer Information and Engineering, Jiangxi Normal University, Nanchang 330022, China; myu821@163.com

**Keywords:** indoor navigation, location, Kinect, IMU, Kalman filters

## Abstract

As indoor mobile navigation suffers from low positioning accuracy and accumulation error, we carried out research into an integrated location system for a robot based on Kinect and an Inertial Measurement Unit (IMU). In this paper, the close-range stereo images are used to calculate the attitude information and the translation amount of the adjacent positions of the robot by means of the absolute orientation algorithm, for improving the calculation accuracy of the robot’s movement. Relying on the Kinect visual measurement and the strap-down IMU devices, we also use Kalman filtering to obtain the errors of the position and attitude outputs, in order to seek the optimal estimation and correct the errors. Experimental results show that the proposed method is able to improve the positioning accuracy and stability of the indoor mobile robot.

## 1. Introduction

In mobile robot navigation, stable and reliable positioning results are the key prerequisite for planning paths. In recent years, Kinect has increasingly been applied in robot obstacle avoidance [1,2], target reconstruction [3,4], target tracking [5,6], attitude control [7,8] and other fields due to its advantageous features. Kinect is a novel 3D stereoscopic camera developed by Microsoft, which can provide RGB and depth information of the mobile robot’s environment for users, and is low in price, making it suitable for replacing conventional ultrasonic radar and laser radar as a distance sensor. Additionally, the acquired environment depth value is continuous, contains a large amount of information, is minimally influenced by light and other features, and can be used in limited-cost positioning situations with certain accuracy requirements. 

Kinect is a visual sensor, and has limitations with respect to accuracy [9] and speed [10,11]. In terms of accuracy, there is a positioning error in the visualization positioning method due to the uncertain location estimation of the spatial feature points. 

Take the visual odometer as an example: the position and attitude of the robot is cumulatively calculated based on the change in position between the current frame and the previous one, meaning that the accumulation of estimation errors per frame will reduce the positioning accuracy as the number of frames increases [12]. 

In extreme cases, feature point extraction and matching algorithms may fail in purely visual autonomous navigation due to insufficient or excessive external light. While in terms of speed, due to the huge amount of image data collected per frame, complicated processing algorithms and lower degrees of parallelism of the corresponding image mean that the speed can not be improved, thus limiting real-time response to positioning accuracy requirements. In contrast, IMU, which has simple, real-time and all-weather completely autonomous navigation functionality, can compensate for visual measurement errors in accuracy and speed [13], although it still has many serious problems, such as accumulated positioning error.

Therefore, this paper investigated indoor mobile robot integrated navigation and positioning based on Kinect and IMU. For high-precision positioning, we firstly use feature point extraction, match the RGB image between the target frame obtained by Kinect and the reference frame, implement the Random Sample Consensus (RANSAC) algorithm to remove the mismatching points, and then use the absolute orientation algorithm to obtain the Kinect posture and offset (here, Kinect pose represents the pose of the robot). Thereby, we obtain the trajectory of mobile robot movement. The visual positioning results and the inertial navigation system (INS) data are fused by Kalman filter algorithm to improve the self-positioning accuracy of the indoor mobile robot. The detailed design process is described in the following sections.

## 2. Independent Localization Based on Kinect and INS

### 2.1. Kinect Method

#### 2.1.1. Kinect Obtaining 3D Point Cloud Data

The pixel coordinates of the feature points in the RGB image coordinates $\left(x_{i},\;y_{i}\right)$ are known, after the angle correction in depth and RGB image, the three-dimensional coordinates of a point in the Kinect v1 coordinate system can be obtained from the depth of the image through the calculation of Equation (1):

(1) $$\begin{array}{c} x_{c}=\left(x_{i}-u_{0}\right)*z/f_{x}\\ y_{c}=\left(y_{i}-v_{0}\right)*z/f_{y}\\ z_{c}=z \end{array}$$

where *z* is the depth value of a feature point, and $u_{0}$, $v_{0}$, $f_{x}$ and $f_{y}$ are the internal orientation elements of the Kinect calibrated RGB camera. The sensor Kinect operates (runs) usually at a range from 0.5 m to 4 m.

#### 2.1.2. Absolute Orientation Algorithm

In order to improve the image of the absolute orientation accuracy of the digital close-range accuracy image, Nanshan Zheng et al. [14] describe an absolute orientation method for close-range images based on the successive correlative condition adjustment model. If the deformation of the model itself is not considered, an absolute orientation model is a spatial similarity conversion issue, including the rotation and displacement of the model coordinate system relative to the target coordinate system, and the scale factor of the model scale. The basic relation of the absolute orientation is shown in Equation (2):

(2) $$\left(\begin{array}{c} X\\ Y\\ Z \end{array}\right)=\lambda \left(\begin{array}{ccc} a_{1} & b_{1} & c_{1}\\ a_{2} & b_{2} & c_{2}\\ a_{3} & b_{3} & c_{3} \end{array}\right)\left(\begin{array}{c} U\\ V\\ W \end{array}\right)+\left(\begin{array}{c} \Delta X\\ \Delta Y\\ \Delta Z \end{array}\right)$$

Among them, (U,V,W) are the points in the local reference frame (i.e., the model coordinates) of the corresponding points (X,Y,Z), which are in the International Terrestrial Reference Frame (ITRF) (i.e., the corresponding target coordinates). Seven absolute orientation elements are: the shift of coordinate origin (ΔX,ΔY,ΔZ), scale factor λ, the three rotation angles between the two coordinate systems {Φ,Ω,K}, and *a_i_*, *b_i_*, *c_i_* are the transform coefficients between the model and target coordinate system. Absolute orientation is a method obtaining seven absolute orientation elements with known control points. Suppose $M\left(X_{M},Y_{M},Z_{M}\right)$, $N\left(X_{N},Y_{N},Z_{N}\right)$, $P\left(X_{P},Y_{P},Z_{P}\right)$, $Q\left(X_{Q},Y_{Q},Z_{Q}\right)$ are the initial position coordinates of the Kinect, $m\left(U_{m},V_{m},W_{m}\right)$, $n\left(U_{n},V_{n},W_{n}\right)$, $p\left(U_{p},V_{p},W_{p}\right)$, $q\left(U_{q},V_{q},W_{q}\right)$ are the adjacent initial position coordinates, wherein λ is 1. Putting coordinates of the four same-named points into Equation (3), and using the equations listed at points N,P,Q*,* separately subtracting the equations listed at point *M* to eliminate the shift parameters, the result is:

(3) $$\left[\begin{array}{ccc} U_{N}-U_{M} & V_{N}-V_{M} & W_{N}-W_{M}\\ U_{P}-U_{M} & V_{P}-V_{M} & W_{P}-W_{M}\\ U_{Q}-U_{M} & V_{Q}-V_{M} & W_{Q}-W_{M} \end{array}\right]\;R_{0}=\left[\begin{array}{ccc} X_{N}-X_{M} & Y_{N}-Y_{M} & Z_{N}-Z_{M}\\ X_{P}-X_{M} & Y_{P}-Y_{M} & Z_{P}-Z_{M}\\ X_{Q}-X_{M} & Y_{Q}-Y_{M} & Z_{Q}-Z_{M} \end{array}\right].$$

Among them:

(4) $$R_{0}=\left(\begin{array}{ccc} a_{1}^{0} & b_{1}^{0} & c_{1}^{0}\\ a_{2}^{0} & b_{2}^{0} & c_{2}^{0}\\ a_{3}^{0} & b_{3}^{0} & c_{3}^{0} \end{array}\right)$$

where *R_0_* is the transformation matrix transforming between the model (the local reference frame) and the target coordinate system (ITRF). The initial rotation angle of the absolute orientations are:

(5) $$\begin{array}{c} \Phi =-\arctan\left(a_{3}^{0}/c_{3}^{0}\right)\\ \Omega =-\arcsin\left(b_{3}^{0}\right)\\ K=\arctan\left(b_{1}^{0}/b_{2}^{0}\right) \end{array}$$

After obtaining the scale factor and the initial rotation angle, the shift parameter (ΔX,ΔY,ΔZ) can be obtained by substituting arbitrary points into the equation. 

#### 2.1.3. Implementation of Kinect Self-Localization Algorithm 

The scale-invariant feature transform (SIFT) matching algorithm [15] has a strong matching ability, and can match the image feature points when any two images are translated, rotated and affine transformed. In this paper, we mainly use SIFT matching, the RANSAC algorithm [16], and the absolute orientation algorithm to realize the robot self-localization scheme. In this section, a method is proposed for how to use the above algorithms to solve the robot autonomous navigation problem. This method consists of four parts: same-named point extraction, abnormal point elimination, region selection, motion parameter calculation.

Same-named point extraction: Obtain the SIFT matching points of two adjacent images from the RGB image, and then obtain the 3D coordinates of all matching point pairs from the depth image. The specific steps include using the SIFT method to extract the common key points in Image1 and Image2 first, and then obtaining the pixel coordinates of SIFTData1 and SIFTData2 in the respective RGB images.

Although the SIFT algorithm is able to extract stable feature points, there will still be some mismatching points. In order not to affect the calculation and improve the accuracy of parameters, the RANSAC algorithm is used to eliminate mismatch points in the initial matching point pair. Then the 3D coordinates of Depth1 and Depth2 of the key points are obtained from the depth images by using new positional information of Data1 and Data2.

Since Kinect is utilizes an infrared camera, it is easily influenced by noise, such as strong light, high density in the layout of feature points, and other factors, which can cause deviations in the depth information obtained by the Kinect, reducing positioning accuracy. Hence, one can use the bubble sorting method to select four intervals of relatively large feature points (generally the area formed by selected points accounting for more than 50% of the entire color image) for the absolute orientation calculation, and take the 3D coordinates of the points around these four feature points, summed and averaged together, thereby improving the accuracy of the motion parameters calculated by the absolute orientation method.

After removing the mismatching points and using regional screening, remaining at least two pairs of high-precision matching points, and so the reliability of the data is increased. 

**Figure 1:**
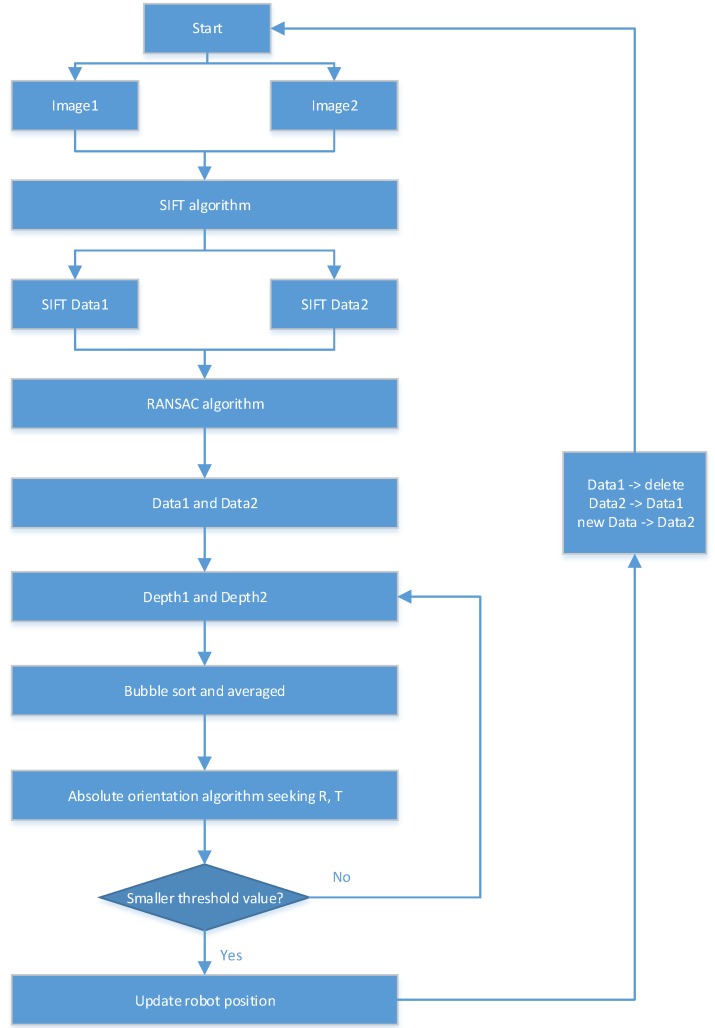
Flowchart of the robot self-localization method.

Assuming the feature point set 3D coordinates acquired by the Kinect in the first position is Data1, and the set acquired in the second position is Data2, combining the coordinates of Data1 and Data2, one can use the absolute orientation algorithm to calculate the rotation matrix and the offset vector in these two positions. Setting the robot’s initial position as the origin of the coordinates, when the robot moves to the third position, the feature point set 3D coordinates acquired by the Kinect in the second position are used as the new Data1, and those from the third position as the new Data2. The new Data1 and Data2 coordinates are used to calculate the relative motion parameters from the second to the third position of the robot, and the trajectories of the robot motion can be obtained by iteration. The flowchart is shown in Figure 1.

### 2.2. Principle and Algorithm Design of SINS

The trapdown Inertial Navigation System (SINS) algorithm [17,18] uses a mathematical platform, and the core part of it is the attitude updating solution. As the angular velocity vector is not a real vector, integrating the angular velocity will produce a noncommutativity rotation error. Thus, the equivalent rotation vector method is used [19]. Due to the superiority of the quaternion algorithm, a quaternion-based rotation vector algorithm is used. Considering that the accuracy and the amount are not high, a three-component algorithm is used to process the equivalent rotation vector problem.

The basic idea of SINS updating is to take the previous navigation parameters (attitude, velocity and position) as initial values, and to calculate the current navigation parameters by using the outputs (angular velocity and acceleration) of the inertial device from the previous time to the current time. Then it takes recursively the current time as the initial values for the next moment calculation. The basic formulas are as follows.

Velocity update algorithm:

(6) $$v_{m}^{n}=v_{m-1}^{n}+F_{v\to q}\left(\frac{-\omega_{inm-1}^{n}T_{m}}{2}\right)q_{bm-1}^{n}\Delta v_{sfm}+\left[g_{m-1}-\left(2\omega_{iem-1}^{n}+\omega_{enm-1}^{n}\right)\times v_{m-1}^{n}\right]T_{m}$$

(7) $$\Delta v_{sfm}=\Delta v_{m}+0.5\Delta \theta_{m}\times \Delta v_{m}+\left\{\left[\sum_{i=1}^{n-1}k_{i}\Delta \theta_{m}\left(i\right)\times \Delta v_{m}\left(n\right)\right]+\left[\sum_{i=1}^{n-1}k_{i}\Delta v_{m}\left(i\right)\right]\times \Delta \theta_{m}\left(n\right)\right\}$$

Location update algorithm:

(8) $$L_{m}=L_{m-1}+\frac{T_{m}v_{Nm,\;m-1}^{n}}{R_{M}}$$

(9) $$\lambda_{m}=\lambda_{m-1}+\frac{T_{m}\sec L_{m-1}v_{Em,\;m-1}^{n}}{R_{N}}$$

(10) $$h_{m}=h_{m-1}+T_{m}v_{Um,\;m-1}^{n}$$

Attitude update algorithm:

(11) $$q_{bm}^{n}=q_{bm-1}^{n}F_{v\to q}\left(\Phi_{m}-q_{bm-1}^{n}*\omega_{inm-1}^{n}T_{m}\right)$$

where *Lm* is the longitude, *λm* is the latitude, *hm* is the height, $T_{m}$ is the sampling period, $\Phi_{m}$ is equivalent rotation matrix, * is common rail, and $\Delta v_{sfm}$ is the speed compensation amount caused by the force (Sculling compensation term), as the gyroscope outputs are the signals of angular increment and velocity increment, they cannot be calculated directly. Equation (7) is a quadratic fitting of the angular rate and acceleration; that is, a three-subsample algorithm.

As the SINS posture updating with a coning motion, it will lead to a serious drift of the mathematical platform, so it is necessary to further optimize the rotation vector algorithm [20,21]. The following formula is the rotation vector optimized by the three-subsample algorithm.

(12) $$\Phi_{m}=\Delta \theta_{1}+\Delta \theta_{2}+\Delta \theta_{3}+\frac{9}{20}\Delta \theta_{1}\times \Delta \theta_{3}+\frac{27}{40}\Delta \theta_{2}\times \left(\Delta \theta_{3}-\Delta \theta_{1}\right)$$

The longitude, latitude, and height have to be transformed into the local coordinate system. For a coordinate transformation from the International Terrestrial Reference Frame (ITRF), such as the World Geodetic System (WGS) 84, to the local coordinate system, several points with the coordinates of both systems were calculated to support the indoor positioning using a local coordinate system.

## 3. Integrated Navigation Scheme

Simple visual navigation will have a cumulative error over a long time, and Global Positioning System (GPS) loses signal indoors and cannot be positioned, so it is necessary to introduce other navigation systems to amend traditional navigation systems. In conclusion, a Kalman filter integrated navigation system based on Kinect/IMU is used to reduce the visual error, so that the positioning accuracy of mobile robots in indoor autonomous navigation is improved.

The Kalman filter [22] is an optimal linear estimation method, which uses the state-space description method to describe the system and makes continuous estimations through the estimation errors of various sensor outputs to optimally combine the information of all the sensors. This optimally estimated value minimizes the linear quadratic loss of the estimation error, including the mean square error of any linear combination of state estimation errors. Note that the Kalman gain is the optimal weighting matrix.

The Kalman filter includes time updating and measurement updating. Measurement updating is performed by measuring the new information obtained from the sensor in order to update the estimated value and error. Time updating mainly considers the uncertain dynamic image, update estimation and estimation error. Since the used state equation is based on the errors, this paper does not deal with the extended Kalman filter. A filter that constitutes a feedback pulse is used here. Basic equations of the discrete Kalman filter are shown below.

One-step state prediction equation:

(13) $${\hat{X}}_{k|k-1}=\Phi_{k|k-1}{\hat{X}}_{k-1}$$

where ${\hat{X}}_{k-1}$ is the system state estimation value at $t_{k-1}$ moment, ${\hat{X}}_{k|k-1}$ is the system state predictive value at $t_{k}$.

State estimation equation:

(14) $${\hat{X}}_{k}={\hat{X}}_{k|k-1}+K_{k}(Z_{k}-H_{k}{\hat{X}}_{k|k-1})$$

Filter gain equation:

(15) $$K_{k}=P_{k|k-1}H_{k}^{T}{\left[H_{k}P_{k|k-1}H_{k}^{T}+R_{k}\right]}^{-1}$$

One-step prediction MSE (mean square error):

(16) $$P_{k|k-1}=\Phi_{k|k-1}P_{k-1}\Phi_{k|k-1}^{T}+\Gamma_{k|k-1}Q_{k}\Gamma_{k|k-1}^{T}$$

Estimated MSE (mean square error):

(17) $$P_{k}=\left[I-K_{k}H_{k}\right]P_{k|k-1}{\left[I-K_{k}H_{k}\right]}^{-1}+K_{k}R_{k-1}K_{k}^{T}$$

**Figure 2:**
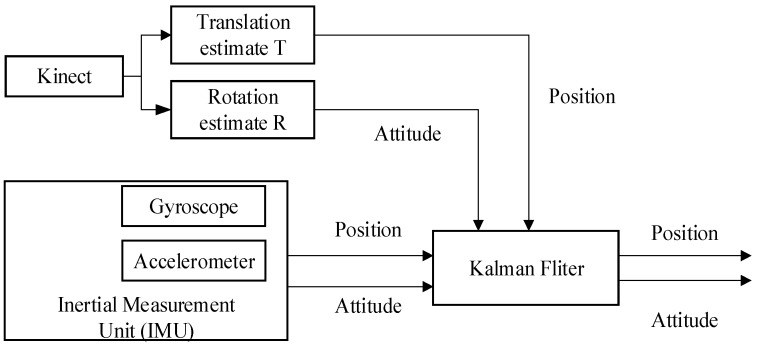
System and filter structure.

Using the Kalman filter linear recursive algorithm to calculate the next moment measurement and state estimates, the initial value of ${\hat{X}}_{0}$ and $P_{0}$ should be known first. The designed Kalman filter model is used to fuse the attitude and position information of the Kinect and IMU, and can be used to modify the trajectory of IMU and make up for errors accumulating over time, thus improving the self-localization accuracy of the indoor robot. The system and filter structure are shown in Figure 2.

## 4. Indoor Positioning Experiment

**Figure 3:**
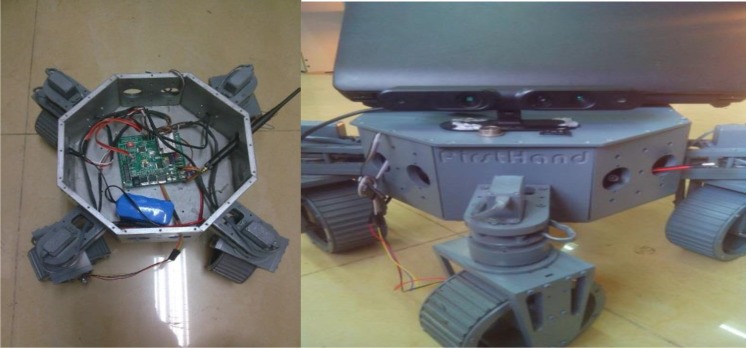
Kinect and experimental platform WX-DP203.

In order to verify the positioning accuracy of the Kalman filter after fusion, an indoor experiment was designed in the study room of our institute, in which the robot went along the wall of the room. Using a WX-DP203 (designed and developed by our navigation group) mobile robot installed with a Kinect sensor and IMU, the robot tracked a wall and landmarks, as shown in Figure 3. Kinect image acquisition frequency is 1 Hz, IMU data collection frequency is 100 Hz. In order to compare the final positioning accuracy, we preplanned the relevant path, set up three control points including the start and the end, and obtained the exact positions of the control points under the local coordinate system by using the total station. With the starting point coordinate set as 0, the measured control point coordinates are shown in Table 1.

**Figure 4:**
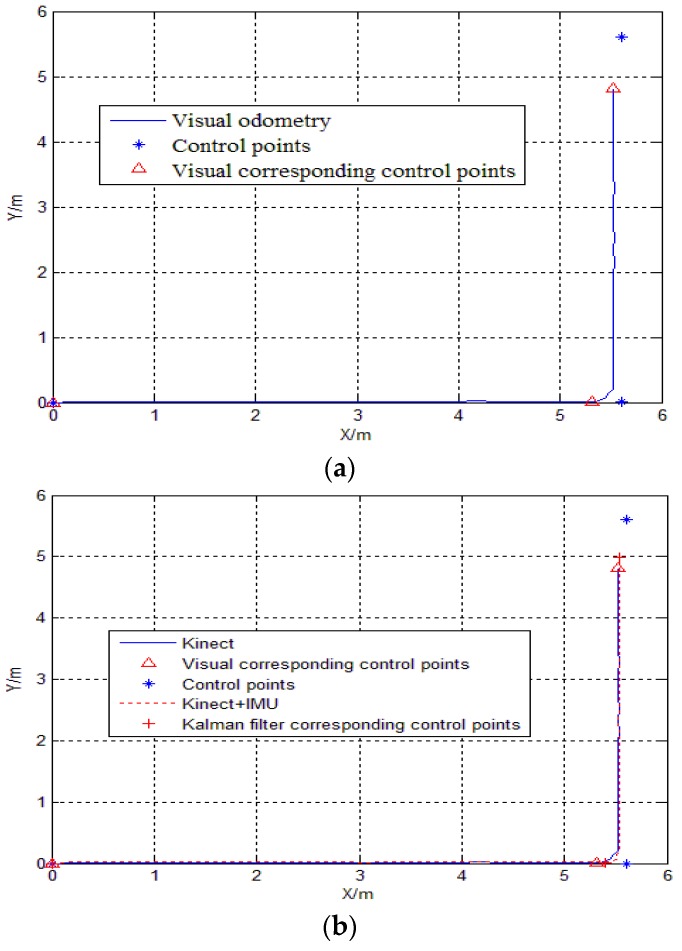
(**a**) Kinect positioning results; (**b**) Comparison of Kalman filter positioning trajectory.

During the experiment, the robot moved 11.2 m, moved no more than 20 cm per second, and obtained 116 frames of color and depth images in total. The results of the pure visual positioning and Kalman filter combination positioning are shown in Figure 4. The corresponding visual positions and the control point positions of the three set points are shown in Table 2, Kalman filter error analysis is shown in Table 3.

It can be seen from Table 2 that when the robot moves in a straight line of 5.61 m, the error accumulates slowly, the positioning accuracy was 0.2890/5.61 × 100% = 5.16%, which is higher. When the robot turns, the error accumulates faster, and the positioning accuracy was 0.7954/5.61 = 14.2%. Considering that the robot itself may sideslip during operation and Kinect sensors are susceptible to noise and other factors, there is some degree of the errors to be expected in the experimental results.

From Table 3, it can be seen that the Kinect/IMU integrated Kalman filter plays a very good role in making up for the cumulative error of the visual range. The combination of these two sensors can achieve higher positioning accuracy. The cumulative error before turning decreased from 0.2890 m to 0.2077 m, the relative visual improved by 1.36%, the cumulative error after turning decreased from 0.7954 m to 0.6078 m, and the relative visual acuity increased by 3.4%. These results show that the cumulative positioning error of the short-distance visual range is very small, while the positioning accuracy is high. We have known that the IMU strapdown solution goes from an unstable to a stable phase in the initial calculation stage, and when a large change in the posture (e.g., a turning along the route) occurs, the strapdown solution will change significantly, and the improvement in the position accuracy after following a short distance is not obvious. However, the accuracy of the orientation angle is better improved, and the stability of the orientation is also improved.

## 5. Conclusions

This paper combined the Kinect sensor with an IMU and the Kalman filter in order to conduct integrated positioning system research. The integrated positioning results of the Kalman filter reduce the trajectory errors of the visual range with an IMU, modify the accumulative IMU errors with the visual range, and improve the positioning accuracy of the indoor robot. The Kinect has the advantages that it utilizes an ordinary RGB camera and depth sensor, and is a cost-effective, simple, and valuable sensor. Since an IMU has been used to increase the data collection numbers with its high sampling rate and relatively high accuracy, the cumulative errors of the integrated system of our method decreased from 0.2890 m to 0.2077 m, and the relative accuracy improved by 1.36% (for accuracy improvement, see the Table 3), compared with the vision system alone. The Kinect and IMU integrated system benefited from each other, which overcame the drawbacks of them, so that improve the system stability. However, the method has still the accuracy limitation, as the research has only just commenced, and much work is still to be done. Our next step is to increase the Kinect measurement range and improve the accuracy of counting motion parameters, as this is easily affected by the noises. In addition, we will try to reduce the swing amplitude when the robot turns, thereby decreasing the attitude errors.

## Figures and Tables

**Table 1:** Control point coordinates (in meters).

Number of Control Points	1	2	3
Control Points	(0, 0)	(5.61, 0.01)	(5.60, 5.61)

**Table 2:** Position comparison of visual and control points (in meters).

Number of Control Points	1	2	3
The position of the control point	(0.00, 0.00)	(5.61, 0.01)	(5.60, 5.61)
Visual position	(0.00, 0.00)	(5.321, 0.0062)	(5.5248, 4.8182)
Distance errors (Positioning errors)	0.00	0.2890	0.7954

**Table 3:** Kalman filter error analysis (in meters).

Number of Control Points	1	2	3
Visual odometry	0.00	0.2890	0.7954
Kalman filter of Kinect/IMU	0.00	0.2077	0.6078
